# Inferring the Provenance of an Alien Species with DNA Barcodes: The Neotropical Butterfly *Dryas iulia* in Thailand

**DOI:** 10.1371/journal.pone.0104076

**Published:** 2014-08-13

**Authors:** Noah A. Burg, Ashman Pradhan, Rebecca M. Gonzalez, Emely Z. Morban, Erica W. Zhen, Watana Sakchoowong, David J. Lohman

**Affiliations:** 1 Sackler Institute for Comparative Genomics, American Museum of Natural History, New York, New York, United States of America; 2 Department of Psychology, Hunter College, City University of New York, New York, New York, United States of America; 3 Biology Ph.D. Program, Graduate Center, City University of New York, New York, New York, United States of America; 4 Biology Department, City College of New York, City University of New York, New York, New York, United States of America; 5 High School for Environmental Studies, New York, New York, United States of America; 6 Department of National Parks, Wildlife and Plant Conservation, Chatuchak, Bangkok, Thailand; 7 Entomology Section, National Museum of the Philippines, Manila, Philippines; Onderstepoort Veterinary Institute, South Africa

## Abstract

The Neotropical butterfly *Dryas iulia* has been collected from several locations in Thailand and Malaysia since 2007, and has been observed breeding in the wild, using introduced *Passiflora foetida* as a larval host plant. The butterfly is bred by a butterfly house in Phuket, Thailand, for release at weddings and Buddhist ceremonies, and we hypothesized that this butterfly house was the source of wild, Thai individuals. We compared wing patterns and COI barcodes from two, wild Thai populations with individuals obtained from this butterfly house. All Thai individuals resemble the subspecies *D. iulia modesta*, and barcodes from wild and captive Thai specimens were identical. This unique, Thai barcode was not found in any of the 30 specimens sampled from the wild in the species' native range, but is most similar to specimens from Costa Rica, where many exporting butterfly farms are located. These data implicate the butterfly house as the source of Thailand's wild *D. iulia* populations, which are currently so widespread that eradication efforts are unlikely to be successful.

## Introduction

The introduction of exotic species to novel habitats is one of the most significant threats to biodiversity conservation. Introduced plants can become invasive, replacing natural vegetation. Introduced predators can consume indigenous prey that lack suitable defenses, and introduced insect herbivores can become plant pests, causing damage to native plants as well as crops [1]. Extensive import laws and quarantine procedures exist in nearly every country to curtail unintentional introduction of pestiferous insects, which could potentially “hitchhike” on imported plants or agricultural produce. Despite the tremendous effort spent trying to prevent the spread of insects between countries, introductions of insect pests are common [1], [2].

Once a newly introduced insect has been discovered, it may not possible to determine how the introduction occurred or whether the same species was introduced multiple times. For example, Eastwood and colleagues [3] used DNA barcodes to demonstrate that all sampled Dominican *Papilio demoleus* shared a single barcode also found throughout most of Southeast Asia. Since the particular barcode haplotype found in the Dominican Republic is widespread in the Oriental Region, it was not possible to determine the precise location of the source population using DNA barcodes or whether the species was introduced more than once [3]. Determining the provenance of this introduction is important because the Southeast Asian lineage of this species is frequently a pest of *Citrus*, whereas the lineage from Australia and New Guinea is not [4], [5]. In addition to assessing the potential crop damage an introduced insect species may cause, knowledge of a species' home range might also be useful for identifying suitable parasitoid species for biological control.

**Figure 1:**
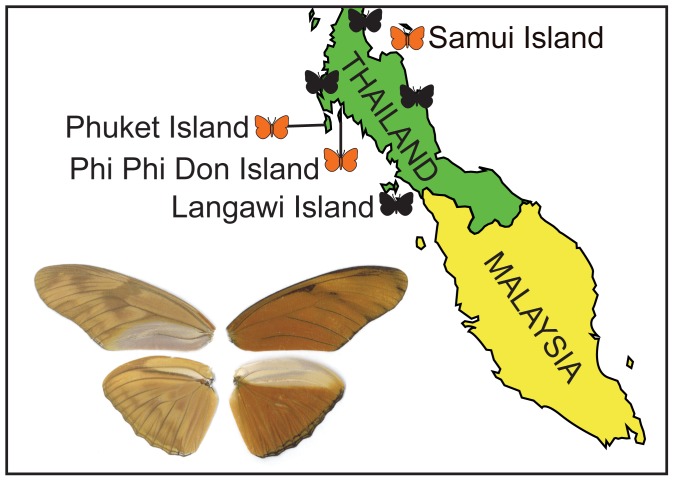
Collection localities of *Dryas iulia* butterflies on the Thai-Malay peninsula (unpublished data) [6], [7]. Orange butterfly symbols indicate localities from which we sampled specimens for this study; black symbols indicate unsampled localities from which the species has been recorded. The image illustrates the wings of specimen DL-08-T033 caught on Phi Phi Don Island, Krabi Province, Thailand.

Beginning in 2007, several independent observers recorded specimens of the Julia butterfly, *Dryas iulia* (Fabricius, 1775), at several locations in Thailand and Malaysia (Fig. 1) including Samui Island (Surat Thani province; Les Day, *pers. comm*.), Phuket Island (Phuket province; Sin Khoon Khew, *pers. comm*.), Tioman Island, Malaysia [6], and Phi Phi Don Island, Thailand (Krabi province; DJL, *pers. obs*.). Küppers [7] reported the species from the Thai provinces of Nakhon Si Thammarat, Phang Nga, and Chumphon, and suggested that the species might have escaped from a butterfly house on Phuket.

*Dryas iulia* is native to the Americas, where thirteen subspecies are found in the southern USA, Central America, the Caribbean, and northern South America [8], [9]. To identify the Thai specimens to subspecies (Fig. 1), we compared wing patterns of this material to published photographs of all subspecies [9]–[11]. Specimens from Thailand resemble the subspecies *Dryas iulia modesta*, found in Texas, Mexico, Central America, and the Pacific coast of South America to Ecuador [8]. In their native range, larvae of *D. iulia* feed on a variety of different *Passiflora* species (Passifloraceae). Plants in this genus are typically vines or lianas, with more than 100 species in the New World tropics, and about 20 in tropical Southeast Asia, Australia, and New Zealand [12]. The second edition of *Butterflies of Thailand* [13] now lists the species as being part of the country's fauna, noting its presence in Chumpon, Nakhon Si Thammarat, and Surat Thani provinces, which are all in the southern peninsula. In Thailand, larvae have been found feeding on *Passiflora foetida*, and adults frequently nectar on *Lantana camara* (Les Day, *pers. comm*.); both of these plants are invasive species native to the Americas [14], [15].

The Phuket Butterfly Garden (PBG; 71/6 Moo 5, Soi Paneung, Yaowarat Road, Rassada Rd., Phuket City) has been open since 1990 in the center of Phuket Island, one of the country's most visited tourist areas. In addition to maintaining a butterfly vivarium, the company sells live butterflies for release at weddings (phuketbutterfly.com/wedding.php, accessed March 2014) and provides butterflies for mass public release [16], [17]. Releasing butterflies at weddings is a relatively new custom practiced around the world. Instead of throwing rice or birdseed at newlyweds as they leave the wedding ceremony, celebrants release live butterflies from an envelope or cage so the couple departs in a swarm of live insects [18], [19]. After noticing that the PBG website (phuketbutterfly.com, accessed May 2008) showed pictures of *D. iulia* butterflies, a Thai colleague visited the facility at our request in June 2008. He found *D. iulia* flying in the vivarium, confirmed with staff that *D. iulia* could be purchased for release at weddings, confirmed that specimens could be shipped to the resort islands of Samui and Phi Phi Don (where *D. iulia* has already been observed in the wild), and obtained nine fresh specimens of this species. We subsequently froze the specimens for genetic work.

In the present study, we address two questions: 1) Did wild Thai populations of *Dryas iulia* originate from livestock at the Phuket Butterfly Garden (PBG)? and 2) From where in its natural range did PBG animals originate? To answer these questions, we sequenced the barcoding section of the mitochondrial *cytochrome c oxidase* subunit I (COI) gene from wild-caught specimens in Thailand and specimens obtained from PBG. COI evolves rapidly, is easily amplified and sequenced with highly conserved primers, and is therefore a good marker for assessing maternal relatedness and, potentially, species membership [20]. If wild Thai *D. iulia* were naturalized after introduction from PBG, then DNA barcodes from wild-caught specimens would be similar or identical to barcodes from specimens obtained from PBG. Genetic differences between wild and PBG-derived specimens would suggest that PBG is not the source of Thailand's naturalized *D. iulia*. However, shared barcode sequences might also result if different populations of the species do not vary at this locus. Therefore, we compared these Thai sequences to barcodes from *D. iulia modesta* specimens sampled throughout the species' native range, including sequences from GenBank and from *D. iulia hispaniola* specimens wild-caught in the Dominican Republic. We suspect that PBG stock originated in Costa Rica, as many Neotropical butterfly farms are found here (Michael Boppré, *pers. comm*.) [18].

## Materials and Methods

### Specimen acquisition

Butterfly specimens were caught with an aerial net in the field. Each specimen's wings were removed from its body. Wings were stored in glassine envelopes and bodies were placed in vials of 100% ethanol and frozen. All specimens collected for this study are vouchered in the DNA and Tissues Collection of the Museum of Comparative Zoology, Harvard University (Table 1). Permission to conduct research in Thailand was granted by the National Research Council of Thailand. Permission to export specimens was granted by the CITES Office of the Department of National Parks, Wildlife and Plant Conservation. Permission to conduct research in the Dominican Republic was granted by the Ministerio de Medio Ambiente y Recursos Naturales. Permission to export specimens was granted by the Secretaria de Estado de Agricultura, Departamento de Vida Silvestre, Santo Domingo. All permits in the Dominican Republic were arranged by Kelvin A. Guerrero (kguerrero.net).

**Table 1:** Specimen information for sequences included in this analysis.

Species	Location: Voucher #	Coll. Date	Coordinates	Collection Locality	GenBank
*Dryas iulia hispaniola*	MCZ: NC-11-J036	22-Apr-11	19.0676 N 70.8639 W	Dominican Republic: La Vega, La Cienaga	KJ496350
*Dryas iulia hispaniola*	MCZ: NC-11-J053	23-Apr-11	19.0969 N 70.6186 W	Dominican Republic: La Vega, Salto Baiguate	KJ496351
*Dryas iulia modesta*	DHJ: 04-SRNP-56202	1-Jun-08		Costa Rica	GU157122
*Dryas iulia modesta*	DHJ: 04-SRNP-56042			Costa Rica	GU157127
*Dryas iulia modesta*	DHJ: 05-SRNP-58644			Costa Rica	GU157128
*Dryas iulia modesta*	DHJ: 05-SRNP-32411			Costa Rica	GU157129
*Dryas iulia modesta*	DHJ: 05-SRNP-45212			Costa Rica	GU157130
*Dryas iulia modesta*	DHJ: 05-SRNP-32379			Costa Rica	GU157131
*Dryas iulia modesta*	DHJ: 08-SRNP-55873	5-May-08	10.763 N 85.413 W	Costa Rica	GU666775
*Dryas iulia modesta*	DHJ: 08-SRNP-72539	10-Oct-08	10.997 N 85.397 W	Costa Rica	GU666782
*Dryas iulia modesta*	DHJ: 07-SRNP-45965			Costa Rica	JQ536893
*Dryas iulia modesta*	DHJ: 03-SRNP-11685	27-Jul-03	10.9066 N 85.2878 W	Costa Rica: Alajuela, Area de Conservacion Guanacaste, Rincon Rainforest, Sendero Juntas	GU333908
*Dryas iulia modesta*	DHJ: 03-SRNP-16486	18-Jul-03	11.0182 N 85.4502 W	Costa Rica: Guanacaste, Area de Conservacion Guanacaste, Del Oro, Tangelo	GU333906
*Dryas iulia modesta*	DHJ: 04-SRNP-49035	7-Oct-04	10.89 N 85.48 W	Costa Rica: Guanacaste, Area de Conservacion Guanacaste, Sector Cacao, Quebrada Otilio	GU157124
*Dryas iulia modesta*	DHJ: 04-SRNP-47806	22-Aug-04	10.89 N 85.48 W	Costa Rica: Guanacaste, Area de Conservacion Guanacaste, Sector Cacao, Quebrada Otilio	GU157126
*Dryas iulia modesta*	DHJ: 04-SRNP-49966	1-Dec-04	10.886 N 85.482 W	Costa Rica: Guanacaste, Area de Conservacion Guanacaste, Sector Cacao, Sendero Guayabal	GU157123
*Dryas iulia modesta*	MAL-02661	26-Aug-99	18.731 N 89.395 W	Mexico: Campeche, Calakmul, Reserva de la Biosfera de Calakmul, 24 Km N X-Pujil, Entrada a ‘El Papagayo’	GU659625
*Dryas iulia modesta*	MAL-02660	14-Aug-02	17.972 N 89.358 W	Mexico: Campeche, Calakmul, Reserva de la Biosfera de Calakmul, Zona K, Dos Naciones	GU659624
*Dryas iulia modesta*	MLL-00747	21-Nov-06	18.68 N 89.40 W	Mexico: Campeche, Calakmul, Zoh Laguna	JN201279
*Dryas iulia modesta*	MLL-01591	29-Mar-07	18.68 N 89.39 W	Mexico: Campeche, Calakmul, Zoh Laguna	JN201280
*Dryas iulia modesta*	MAL-02658	14-May-04	18.484 N 89.899 W	Mexico: Campeche, Ejido Conhuas, Campamento Yax'che, Guardaraya Sur	GU659691
*Dryas iulia modesta*	MAL-02696	9-Jul-02	18.608 N 89.846 W	Mexico: Campeche, Ejido Conhuas: camino a la zona arqueologica Nadzcaan	GU659594
*Dryas iulia modesta*	MAL-02664	23-Feb-99	18.253 N 89.451 W	Mexico: Campeche, Ejido Narciso Mendoza	GU659620
*Dryas iulia modesta*	MAL-02664	23-Feb-99	18.253 N 89.451 W	Mexico: Campeche, Ejido Narciso Mendoza	GU659622
*Dryas iulia modesta*	MAL-02663	3-Jan-93	21.204 N 86.713 W	Mexico: Quintana Roo, Isla Mujeres	GU659619
*Dryas iulia modesta*	MAL-02659	3-Nov-04	19.157 N 87.544 W	Mexico: Quintana Roo, Reserva de la Biosfera Sian ka'an: Camino a Tampalam	GU659623
*Dryas iulia modesta*	MAL-02698	11-Mar-04	19.722 N 87.812 W	Mexico: Quintana Roo, Reserva de la Biosfera Sian ka'an: Estacion Santa Teresa	GU659588
*Dryas iulia modesta*	MAL-02662	14-Aug-95	18.367 N 88.585 W	Mexico: Quintana Roo, Sabidos	GU659626
*Dryas iulia modesta*	MAL-02665	20-Jan-07	21.344 N 87.63 W	Mexico: Yucatan, Tizimin, La Florida	GU659621
*Dryas iulia modesta*	YB-BCI1550			Panama	HM416486
*Dryas iulia modesta*	MCZ: DL-08-T033	11-Feb-08	7.7611 N 98.7703 E	Thailand: Krabi, Koh Phi Phi Don	KJ496352
*Dryas iulia modesta*	MCZ: PBG-08-D001	1-Jun-08	7.8834 N 98.3956 E	Thailand: Phuket, Phuket City (Ampuh Mueng), Phuket Butterfly Garden	KJ496359
*Dryas iulia modesta*	MCZ: PBG-08-D002	1-Jun-08	7.8834 N 98.3956 E	Thailand: Phuket, Phuket City (Ampuh Mueng), Phuket Butterfly Garden	KJ496360
*Dryas iulia modesta*	MCZ: PBG-08-D004	1-Jun-08	7.8834 N 98.3956 E	Thailand: Phuket, Phuket City (Ampuh Mueng), Phuket Butterfly Garden	KJ496361
*Dryas iulia modesta*	MCZ: PBG-08-D005	1-Jun-08	7.8834 N 98.3956 E	Thailand: Phuket, Phuket City (Ampuh Mueng), Phuket Butterfly Garden	KJ496362
*Dryas iulia modesta*	MCZ: PBG-08-D006	1-Jun-08	7.8834 N 98.3956 E	Thailand: Phuket, Phuket City (Ampuh Mueng), Phuket Butterfly Garden	KJ496363
*Dryas iulia modesta*	MCZ: PBG-08-D007	1-Jun-08	7.8834 N 98.3956 E	Thailand: Phuket, Phuket City (Ampuh Mueng), Phuket Butterfly Garden	KJ496364
*Dryas iulia modesta*	MCZ: PBG-08-D008	1-Jun-08	7.8834 N 98.3956 E	Thailand: Phuket, Phuket City (Ampuh Mueng), Phuket Butterfly Garden	KJ496365
*Dryas iulia modesta*	MCZ: PBG-08-D009	1-Jun-08	7.8834 N 98.3956 E	Thailand: Phuket, Phuket City (Ampuh Mueng), Phuket Butterfly Garden	KJ496366
*Dryas iulia modesta*	MCZ: PBG-08-D010	1-Jun-08	7.8834 N 98.3956 E	Thailand: Phuket, Phuket City (Ampuh Mueng), Phuket Butterfly Garden	KJ496367
*Dryas iulia modesta*	MCZ: LD-08-A15	1-Apr-08	9.5 N 100 E	Thailand: Surat Thani, Koh Samui	KJ496353
*Dryas iulia modesta*	MCZ: LD-08-A17	1-Apr-08	9.5 N 100 E	Thailand: Surat Thani, Koh Samui	KJ496354
*Dryas iulia modesta*	MCZ: LD-08-A19	1-Apr-08	9.5 N 100 E	Thailand: Surat Thani, Koh Samui	KJ496355
*Dryas iulia modesta*	MCZ: LD-08-A20	1-Apr-08	9.5 N 100 E	Thailand: Surat Thani, Koh Samui	KJ496356
*Dryas iulia modesta*	MCZ: LD-08-A21	1-Apr-08	9.5 N 100 E	Thailand: Surat Thani, Koh Samui	KJ496357
*Dryas iulia modesta*	MCZ: LD-08-A22	1-Apr-08	9.5 N 100 E	Thailand: Surat Thani, Koh Samui	KJ496358
*Dryadula phaetusa*	DHJ: 05-SRNP-31045			Costa Rica	GU157119

GenBank accession numbe rs beginning with KJ correspond to novel sequences generated in this study; all other accession numbers represent sequences downloaded from GenBank for inclusion in the analysis. Subspecies identifications of Central American specimens are inferred based on their collection locality. Voucher locations: MCZ = Museum of Comparative Zoology, Harvard University; DHJ = D. H. Janzen collection. Some voucher locations were not specified on GenBank.

### DNA sequencing

Specimens were obtained from colleagues, from the Phuket Butterfly Garden (see Introduction), and from field collection in Thailand and the Dominican Republic. DNA was extracted from single butterfly legs using a QIAGEN DNEasy Blood & Tissue Kit. After addition of the tissue lysis buffer, insect legs were ground mechanically in microcentrifuge tubes using disposable pestles. This step was added to further break down the chitin exoskeleton and thereby maximize the surface area of tissues exposed to the lysis mixture. Subsequently, proteinase-K was added and the manufacturer's protocol was resumed.

A 658 bp fragment of the mitochondrial gene *cytochrome c oxidase* subunit I (COI) was amplified from whole genomic extracts using the diverse metazoan invertebrate primer pair LCO1490 (5′- **TGTAAAACGACGGCCAGT**GGTCAACAAATCATAAAGATATTGG-3′) and HCO2198 (5′-**CAGGAAACAGCTATGAC**TAAACTTCAGGGTGACCAAAAAATCA-3′). These primer sequences include the original primers of Folmer *et al.* [21] to which M13 tails (indicated in bold) had been concatenated on the 5′ end [22]. Addition of these tails to the primers increases PCR success, particularly on specimens with degraded DNA [23]. PCR products were visualized on agarose gels before being sent to Genewiz (genewiz.com) for PCR clean-up and bidirectional sequencing. The primer “tails” M13F and M13R were used as sequencing primers [22]. We sequenced the COI barcode from 18 *D. iulia* specimens, constituting all Thai and Dominican samples in our dataset. We added 28 additional *D. iulia* barcode sequences from Costa Rica, Mexico, and Panama and a sequence from the outgroup *Dryadula phaetusa* (Nymphalidae: Heliconiinae) to the genetic dataset. We included all *Dryas iulia* sequences in GenBank that completely overlapped with the barcoding fragment that we sequenced; longer sequences were trimmed so that each sample included exactly 658 bp. The two Dominican specimens represent the subspecies *D. iulia hispaniola*. All other sequences, including those from Thailand, are of *D. iulia modesta*. Sequences from the other eleven *D. iulia* subspecies—which are mostly Caribbean island endemics—were unavailable. Sequences were viewed, assembled, aligned, and trimmed with Geneious [24]; alignments were performed within Geneious using MUSCLE [25]. The sequence alignment is provided as a nexus file in Appendix S1. Protocols were adopted from dnabarcoding101.org, developed by the DNA Learning Center, Cold Spring Harbor Laboratory.

### Phylogenetic and distance analyses

The most parsimonious haplotype network of *D. iulia* was determined with TCS 1.2 with a 95% connection limit [26], and redrawn using the Pie Graph Tool in Adobe Illustrator CS6 (adobe.com). The program jModelTest 2.1.4 [27] was used to select the GTR+I+G model of sequence evolution using the AIC criterion, but we implemented the GTR+G model to avoid overparameterizing the data. A maximum likelihood analysis and an ML rapid bootstrap analysis were performed with RAxML 7.6.3 [28] on the CIPRES Science Gateway (phylo.org) [29]. Bootstrapping was stopped automatically using the majority rule criterion under a GTR+G model. Bayesian phylogenetic analyses were performed with MrBayes 3.2.2 [30] on the CIPRES Science Gateway. Four Markov chains, one cold and three heated, were run simultaneously for 10 million generations. Trees were sampled every 1000 generations, and the first 25% of sampled trees were discarded as burn-in before calculating a consensus tree. Changes in the posterior probabilities of 20 nodes were plotted over the generations of the analysis with the program *Are We There Yet?* [31] in order to confirm that the chains had probably converged. To assess parsimony support for relationships among taxa, 1000 bootstrap replicates were run in TNT 1.1 using standard bootstrapping with replacement after “Max. trees” was reset to 10,000 [32]. TaxonDNA 1.0 (taxondna.sourceforge.net) [33] was used to calculate uncorrected p-distances between barcode sequences.

## Results

**Figure 2:**
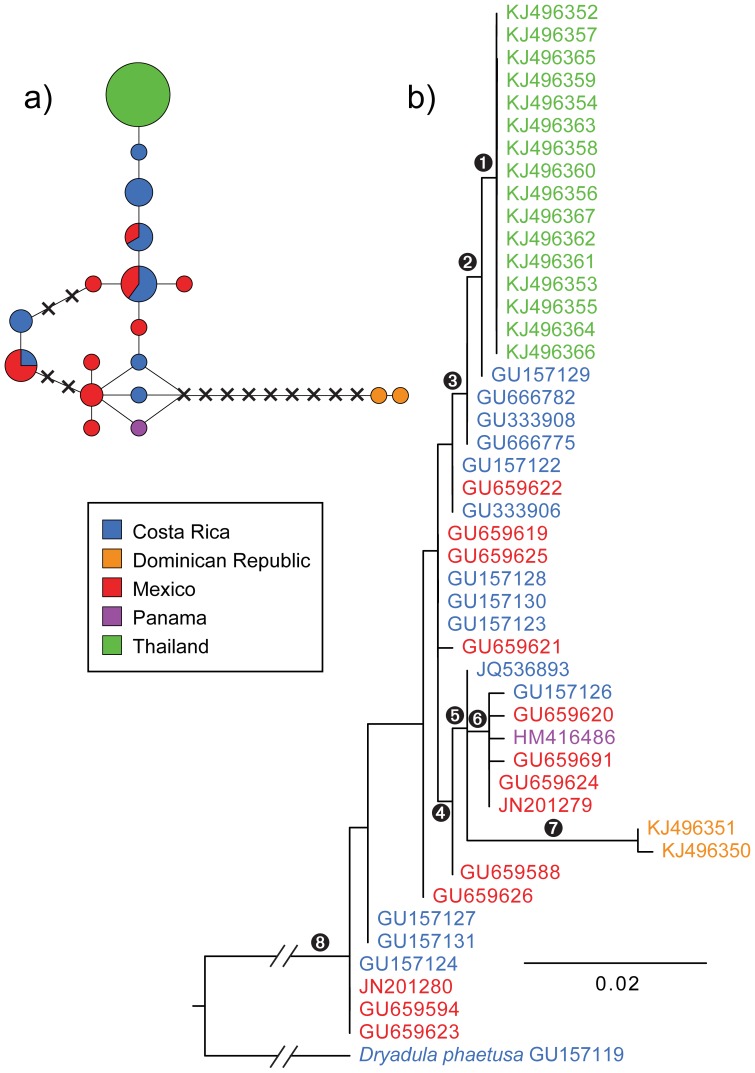
Relationships among *Dryas iulia* showing that wild-caught, Thai specimens have identical barcodes with specimens from the Phuket Butterfly Garden and no other samples collected in Central America and the Caribbean. a) Most parsimonious haplotype network of *D. iulia* constructed with 95% connection limit. The size of each circle is proportional to the number of specimens sharing that haplotype; the smallest circles represent a single haplotype and the largest, sixteen. The colors of the pie charts indicate proportional representation of the provenance of samples with that haplotype. Separation by a line indicates a single base pair difference between haplotypes; crosses represent haplotypes that would be 1 bp different than adjacent haplotypes, but were not sampled in this study. b) maximum likelihood bootstrap consensus tree of COI haplotypes from *D. iulia* and one outgroup. Codes refer to GenBank Accession Numbers and colors denote provenance of specimen collection. Numbers near selected nodes indicate refer to the following branch support values (maximum likelihood bootstrap support, Bayes posterior probability, parsimony bootstrap support, respectively): 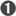 = 40, –, –; 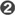 = 27, 0.62, –; 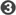 = 20, –, –; 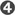 = 27, 0.75, –; 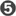 = 31, 0.98, –; 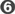 = 26, 0.69, –; 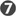 = 100, 1, 100; 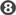 = 100, 1, 100.

DNA barcode sequences were identical among all specimens from Thailand: the single wild-caught *Dryas iulia* on Koh Phi Phi Don, the six wild-caught specimens on Koh Samui, and all nine specimens obtained from the Phuket Butterfly Garden (Fig. 2a). This 658 bp haplotype was not shared with any specimens caught in the New World, but was most similar to a Costa Rican specimen (1 bp difference). The tree topologies obtained from Bayesian, maximum likelihood, and parsimony methods were similar and had universally poor branch support, as one might expect of a phylogeny based on a single gene sampled within a single species (Fig. 2b). Each of the two *D. iulia hispaniola* specimens sampled from the Dominican Republic had a unique haplotype; both were notably distinct from the other sampled haplotypes. Several haplotypes were found in both Mexico and Costa Rica, demonstrating genetic diversity within the subspecies *D. iulia modesta*.

## Discussion

It is likely that wild populations of *Dryas iulia* in Thailand originated from livestock at a butterfly farm, possibly from individuals that were intentionally released alive. One such farm, the Phuket Butterfly Garden (PBG), may be the source of the wild population, but without exhaustive sampling, we cannot rule out other such farms as potential sources of the naturalized wild Thai population. There are at least two alternative scenarios consistent with our results. It is possible that the *D. iulia* livestock at PBG was obtained from the same source as a second, unknown source that was responsible for the introduction—perhaps another butterfly farm in Southeast Asia unknown to us. Alternatively, *D. iulia* could have been introduced into the wild in Thailand where they became established and subsequently collected by PBG for propagation and sale. We consider both of these alternatives unlikely. We know of no other butterfly houses in Thailand that stock non-Asian species, including Nong Nooch Tropical Garden, Pattaya (nongnoochgarden.com), Siam Insect Zoo, Chiang Mai (malaeng.com), Bangkok Butterfly Garden and Insectarium, and Bai Orchid and Butterfly Garden in Chiang Mai. Access to import documentation or knowledgeable PBG staff members could confirm or refute the second possibility.

Identical sequences between wild-caught specimens and those from PBG are not due to lack of genetic diversity in the species or subspecies. We included all publicly available, homologous *D. iulia* barcode sequences in our dataset (which happened to all be from the subspecies *D. iulia modesta*), and the 30 sequences from non-Thai samples constitute 17 distinct haplotypes, demonstrating some degree of genetic variability within the species as a whole. The lack of genetic diversity within Thai *D. iulia* is consistent with a genetic bottleneck caused by a small founding population. This might have happened if a small number of individuals was imported to Thailand and used to found a colony at a butterfly house that eventually became inbred [34].

It is unclear how this novel introduction will affect wild populations of other organisms. The species has been observed feeding on *Passiflora foetida*, which is an invasive plant in Thailand, and the butterfly might therefore be a boon for biological control of this weed. However, herbivory by *D. iulia* might suppress populations of other species through consumptive competition. This vine also provides fodder for the native butterfly species *Cethosia cyane* and *Vindula erota*, as well as the alien species *Acraea terpsicore* (* = A. violae*) [13], which could be adversely affected.

Naturalization of this exotic species in Thailand may not have dire ecological consequences. The Monarch butterfly, *Danaus plexippus*, dispersed across the Pacific Ocean from the Americas to Australia in the 19^th^ century [35], [36]. This relatively recent addition to Australia's biota does not seem to suppress populations of native species, particularly since the larvae feed on introduced plant species including *Asclepias curassavica* and *Gomphocarpus fruticosus* ( = *Asclepias fruticosa*) [5]. Introduced insect species occasionally increase their host breadth to include plant species native to the area of introduction [37], and *D. iulia* might impact native vegetation if this occurs. Observations of the species are currently confined to peninsular Thailand and Malaysia. Wild *D. iulia* was first recorded in Asia only seven years ago, and the species may still be expanding its range. Continued live butterfly release at weddings and religious ceremonies may be fortifying wild populations and aiding range expansion.

There are several ecological dangers associated with butterfly houses. Most of these facilities do not breed butterflies for display. Instead, they are sent shipments of live pupae from butterfly farms by express mail. Many of these shipments cross international borders, as the majority of butterfly farms are in tropical countries and many butterfly houses are in temperate areas [18]. A relatively small number of butterfly farms supply pupae for most of the world's butterfly houses, with large numbers of butterfly farms in Costa Rica, Malaysia, and the Philippines. This translocation of livestock opens the possibility that exotic species could escape into areas where they are not native, thereby introducing novel and potentially pestiferous lepidopteran species into natural ecosystems, as seems to have happened in Thailand. If an escapee is from a species found locally, interbreeding between introduced and native genotypes could disrupt locally co-adapted gene complexes. This insidious “biopollution” of a gene pool could be harmful to species with separate populations that are locally adapted to different conditions. Even if butterflies remain contained within the facilities designed to house them, lepidopteran parasites and pathogens harbored by the living, translocated pupae are smaller and not easily detected. Escape of these butterfly enemies into the wild could have profoundly negative consequences on local butterfly populations [18]. However, it is possible that these risks can be offset to some degree by the potential for butterfly houses to educate the public about basic biology and the importance of wild insects and their habitats, which are threatened around the world [18].

In early 2014, PBG's website showed photographs of at least three different couples in wedding garb releasing butterflies from a cage, and at least one, live *D. iulia* specimen can be seen in each photograph (phuketbutterfly.com/wedding.php, accessed March 2014). In addition to release at weddings, thousands of butterflies are released annually into the Khao Phra Thaeo Wildlife Conservation Area in Phuket in a release ceremony orchestrated in part by PBG [16], [17]. The Phuket Gazette, a local newspaper, has recorded videos of these events in which release of *D. iulia* can be observed [16], [17]. In many parts of Asia, captive animals have been released into the wild for over 1,000 years as part of Buddhist rituals aimed at cultivating compassion for living beings [38]. In recent decades, exotic species are readily available in live animal markets in Asia either as pets or food. Release of these non-native species has led to their establishment as invasive species in some areas [38]–[40]. For example, the American turtle *Trachemys scripta*, which is sold as food and frequently released into the wild, is now the most common turtle in every river in Taiwan [41]. There are several initiatives to educate Buddhist monks and laity about the ecological dangers of animal release [38], [40], [41].

Whereas butterfly houses offer the advantages of conservation awareness and general education about the importance of biodiversity, there are few, if any, positive environmental aspects of intentional butterfly release. Species introduction, biopollution of natural gene pools, and introduction of novel butterfly enemies are all far more likely when fecund, living butterflies are intentionally released into the wild [18], [19]. For these and other reasons, several authors have called for a ban on the release of butterflies at weddings [42]–[45]. Within the United States, USDA-APHIS releases specific guidelines regarding the butterfly species that can be legally released in each state [46] in order to reduce the likelihood of negative ramifications of live butterfly release. We concur with other authors [42]–[45] that the release of live butterflies at social or cultural events should be banned; the short-lived benefits do not justify the threats of long-term damage.

## Conclusions

Our analyses suggest that the Neotropical butterfly *Dryas iulia* was introduced to Thailand by the Phuket Butterfly Garden (PBG), which breeds the species for live release at weddings and other public events. Most wild *D. iulia* locality records in Thailand and Malaysia are on tropical islands that are frequently the site of destination weddings: Phuket, Phi Phi Don, Samui, and Tioman. It is likely that PBG obtained livestock from a butterfly farm in Costa Rica (as evidenced by similarity of barcode sequences), and subsequent inbreeding at the PBG expunged genetic variation, if there was any in the founding population. Released specimens bred in the wild and began using *Passiflora foetida* as a larval host plant. The distribution of the species in Thailand currently encompasses thousands of square kilometers, and eradication efforts are unlikely to be successful, particularly since *P. foetida* is a common, invasive species, making it difficult to find all possible larval host plants for control purposes. To strengthen our conclusions regarding the provenance of the Thai stock, future studies might include more markers and obtain samples from the species' entire native range, which includes most islands of the Caribbean as well as northern South America. We suggest that Thai authorities prohibit the intentional release of live butterflies for commercial purposes and social functions, and regulate the importation of live animals for non-scientific purposes, including insects, to prevent similar introductions in the future.

## Supporting Information

Appendix S1 Nexus file including the DNA sequence alignment analyzed in this paper. Sequence names refer to GenBank accession numbers followed by the two-letter abbreviation for the country where the specimen was collected.
(NEX)

## References

[1] Mooney HA, Hobbs RJ (2000) Invasive Species in a Changing World. Washington, DC: Island Press. 384 p.

[2] Walter GH (2003) Insect Pest Management and Ecological Research. Cambridge: Cambridge University Press. 400 p.

[3] Eastwood R, Boyce SL, Farrell BD (2006) The provenence of old world swallowtail butterflies, *Papilio demoleus* (Lepidoptera: Papilionidae), recently discovered in the new world. Ann Entomol Soc Am 99: 164–168.

[4] Fenner TL, Lindgren E (1974) The life history and larval foodplants of *Papilio demoleus* L. (Lepidoptera: Papilionidae) in southern New Guinea. Papua New Guinea Sci Soc Proc 63–71.

[5] Braby MF (2000) Butterflies of Australia: Their Identification, Biology, and Distribution. Collingwood: CSIRO Publishing. 976 p.

[6] Khew SK (2009) New taxon for Malaysia. Discovery of a new butterfly species on Pulau Langkawi – *Dryas iulia* Available: http://butterflycircle.blogspot.com/2009/09/new-taxon-for-malaysia.html. Accessed 15 February 2014.

[7] Küppers PV (2007) Ist *Dryas iuli*a (Fabricius, 1775) mittlerweile ein fester Bestandteil der thailändischen Lepidopterenfauna? (Lepidoptera, Nymphalidae). Atalanta 38: 325–328.

[8] Clench HK (1975) Systematic notes on *Dryas iulia* (Heliconiidae). J Lepid Soc 29: 230–235.

[9] Warren AD, Davis KJ, Stangeland EM, Pelham JP, Grishin NV (2013) Illustrated Lists of American Butterflies. Available: http://www.butterfliesofamerica.com/. Accessed 15 March 2014.

[10] DeVries PJ (1987) The Butterflies of Costa Rica and their Natural History. Princeton: Princeton University Press. 327 p.

[11] Hernández LR (2004) Field Guide of Cuban-West Indies Butterflies. Maracaibo, Venezuela: Ediluz. 269 p.

[12] Hansen AK, Gilbert LE, Simpson BB, Downie SR, Cervi AC, et al (2006) Phylogenetic relationships and chromosome number evolution in *Passiflora* . Syst Bot 31: 138–150.

[13] Ek-Amnuay P (2012) Butterflies of Thailand: 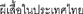 , 2nd Edition. Bangkok: Amarin Publishing.

[14] ISSG (2014) Global Invasive Species Database. Available: http://www.issg.org/database/welcome/. Accessed 28 February 2014.

[15] de Wilde WJJO (1972) Passifloraceae. Flora Malesiana Series 1 7: 405–434.

[16] Anonymous (2012) Phuket butterfly release. Available: http://www.phuketgazette.net/tv/Phuket-Events/Phuket-Butterfly-Release/3262. Accessed 11 May 2014.

[17] Anonymous (2010) Butterflies released. Available: http://www.phuketgazette.net/tv/PGTV-News/Butterflies-Released/1560. Accessed 11 May 2014.

[18] Boppré M, Vane-Wright RI (2012) The butterfly house industry: conservation risks and education opportunities. Conserv Soc 10: 285–303.

[19] New TR (2007) Are butterfly releases at weddings a conservation concern or opportunity? J Insect Conserv 12: 93–95.

[20] Hebert PDN, Cywinska A, Ball SL, deWaad JR (2003) Biological identifications through DNA barcodes. Proc R Soc B 270: 313–321. 10.1098/rspb.2002.2218 PMC1691236 12614582

[21] Folmer O, Black M, Hoeh W, Lutz R, Vrijenhoek R (1994) DNA primers for amplification of mitochondrial *cytochrome c oxidase* subunit I from diverse metazoan invertebrates. Mol Mar Biol Biotech 3: 294–299. 7881515

[22] Messing J (1983) New M13 vectors for cloning. Methods Enzymol 101: 20–78. 6310323 10.1016/0076-6879(83)01005-8

[23] Regier JC, Shi D (2005) Increased yield of PCR product from degenerate primers with nondegenerate, nonhomologous 5′ tails. BioTechniques 38: 34–38. 15679081 10.2144/05381BM02

[24] Drummond AJ, Ashton B, Buxton S, Cheung M, Cooper A, et al. (2009) Geneious v4.6.7 Build 2009-09-22 14:06, Available from http://www.geneious.com/.

[25] Edgar RC (2004) MUSCLE: multiple sequence alignment with high accuracy and high throughput. Nucleic Acids Res 32: 1792–1797. 15034147 10.1093/nar/gkh340 PMC390337

[26] Clement M, Posada D, Crandall KA (2000) TCS: a computer program to estimate gene genealogies. Mol Ecol 9: 1657–1659. 11050560 10.1046/j.1365-294x.2000.01020.x

[27] Darriba D, Taboada GL, Doallo R, Posada D (2012) jModelTest 2: more models, new heuristics and parallel computing. Nature Methods 9: 772–772. 10.1038/nmeth.2109 PMC4594756 22847109

[28] Stamatakis A (2006) RAxML-VI-HPC: Maximum Likelihood-based phylogenetic analyses with thousands of taxa and mixed models. Bioinformatics 22: 2688–2690. 16928733 10.1093/bioinformatics/btl446

[29] Miller MA, Pfeiffer W, Schwartz T (2010) Creating the CIPRES Science Gateway for inference of large phylogenetic trees. Gateway Computing Environments Workshop (GCE). New Orleans. pp. 1–8.

[30] Ronquist F, Teslenko M, van der Mark P, Ayres DL, Darling A, et al (2012) MrBayes 3.2: efficient Bayesian phylogenetic inference and model choice across a large model space. Syst Biol 61: 539–542. 22357727 10.1093/sysbio/sys029 PMC3329765

[31] Nylander JAA, Wilgenbusch JC, Warren DL, Swofford DL (2008) AWTY (are we there yet?): a system for graphical exploration of MCMC convergence in Bayesian phylogenetics. Bioinformatics 24: 581–583. 17766271 10.1093/bioinformatics/btm388

[32] Goloboff PA, Farris JS, Nixon KC (2008) TNT, a free program for phylogenetic analysis. Cladistics 24: 774–786.

[33] Meier R, Shiyang K, Vaidya G, Ng PKL (2006) DNA barcoding and taxonomy in Diptera: a tale of high intraspecific variability and low identification success. Syst Biol 55: 715–728. 17060194 10.1080/10635150600969864

[34] Woodworth L, Montgomery M, Briscoe D, Frankham R (2002) Rapid genetic deterioration in captive populations: causes and conservation implications. Conserv Genet 3: 277–288.

[35] Zalucki MP, Clarke AR (2004) Monarchs across the Pacific: the Columbus Hypothesis revisited. Biol J Linn Soc 82: 111–121.

[36] Vane-Wright RI (1993) The Columbus Hypothesis: an explanation for the dramatic 19th century range expansion of the Monarch Butterflies. In: S. B Malcolm, M. P Zalucki, editors. Biology and Conservation of the Monarch Butterfly. Los Angeles: Natural History Museum of Los Angeles County. pp. 179–187.

[37] Strong DR, Lawton JH, Southwood SR (1984) Insects on Plants: Community Patterns and Mechanisms. Cambridge: Harvard University Press. 311 p.

[38] Shiu H, Stokes L (2008) Buddhist animal release practices: historic, environmental, public health and economic concerns. Contemp Buddhism 9: 181–196.

[39] Corlett R (2010) Invasive aliens on tropical East Asian islands. Biodivers Conserv 19: 411–423.

[40] Liu X, McGarrity ME, Bai C, Ke Z, Li Y (2013) Ecological knowledge reduces religious release of invasive species. Ecosphere 4: 21.

[41] Severinghaus LL, Chi L (1999) Prayer animal release in Taiwan. Biol Conserv 89: 301–304.

[42] Glassberg J, Opler PA, Pyle RM (2014) There's no need to release butterflies—they're already free. Available: http://www.naba.org/weddings.html. Accessed 15 February 2014.

[43] Pyle RM (2010) Under their own steam: the biogeographic case against butterfly releases. News Lepid Soc 52: 54–57.

[44] Pyle RM, Jepsen SJ, Hoffman Black S, Monroe M (2010) Xerces Society Policy on Butterfly Releases. Available: http://www.xerces.org/wp-content/uploads/2010/08/xerces-butterfly-release-policy.pdf. Accessed 15 February 2014.

[45] Kirkwood J (1998) Do commercial butterfly releases pose a threat to wild populations? Available: http://www.nwf.org/News-and-Magazines/National-Wildlife/Animals/Archives/1999/Captive-Butterfly-Threats.aspx. Accessed 28 February 2014.

[46] Wehling W (2012) USDA-APHIS-PPQ Butterfly Environmental Release Decision Chart. Available: http://www.aphis.usda.gov/plant_health/permits/organism/downloads/decision_chart.pdf. Accessed 15 February 2014.

